# PCLPred: A Bioinformatics Method for Predicting Protein–Protein Interactions by Combining Relevance Vector Machine Model with Low-Rank Matrix Approximation

**DOI:** 10.3390/ijms19041029

**Published:** 2018-03-29

**Authors:** Li-Ping Li, Yan-Bin Wang, Zhu-Hong You, Yang Li, Ji-Yong An

**Affiliations:** 1 Department of Information Engineering, Xijing University, Xi’an 710123, China; cs2bioinformatics@gmail.com (L.-P.L.); sxxyliyang@163.com(Y.L.); 2 Xinjiang Technical Institute of Physics and Chemistry, Chinese Academy of Science, Urumqi 830011, China; wangyanbin15@mails.ucas.ac.cn; 3 School of Computer Science and Technology, China University of Mining and Technology, Xuzhou 21116, China; ajy@cumt.edu.cn

**Keywords:** protein–protein interactions (PPI), low rank, protein sequence, relevance vector machine (RVM), evolutionary information

## Abstract

Protein–protein interactions (PPI) are key to protein functions and regulations within the cell cycle, DNA replication, and cellular signaling. Therefore, detecting whether a pair of proteins interact is of great importance for the study of molecular biology. As researchers have become aware of the importance of computational methods in predicting PPIs, many techniques have been developed for performing this task computationally. However, there are few technologies that really meet the needs of their users. In this paper, we develop a novel and efficient sequence-based method for predicting PPIs. The evolutionary features are extracted from the position-specific scoring matrix (PSSM) of protein. The features are then fed into a robust relevance vector machine (RVM) classifier to distinguish between the interacting and non-interacting protein pairs. In order to verify the performance of our method, five-fold cross-validation tests are performed on the *Saccharomyces cerevisiae* dataset. A high accuracy of 94.56%, with 94.79% sensitivity at 94.36% precision, was obtained. The experimental results illustrated that the proposed approach can extract the most significant features from each protein sequence and can be a bright and meaningful tool for the research of proteomics.

## 1. Introduction

Protein–protein interactions (PPI) are a key step in the realization of protein function within cell cycle progression, DNA replication, and signal transmission [1,2,3]. With the development of high-throughput biological technologies, including a yeast two-hybrid screen (Y2H) [4], protein chip technology [5], mass spectrometry [6], and tandem affinity purification tagging (TAP) [7], more PPI data have been accumulated [8]. PPI datasets have been stored in a number of constructed databases, such as the Molecular Interaction database (MINT), the Database of Interacting Proteins (DIP), and the Biomolecular Interaction Network Database (BIND) [8,9,10]. However, experimental methods are labor-intensive and time-consuming. The number of PPIs that are validated by these methods represents only a small portion of the entire PPI network. Moreover, the experimental methods are usually associated with a high rate of both false negative and false positive predictions. All of these drawbacks encourage further research into a computational approach for identifying PPIs.

Different kinds of available protein data are obtained by previous experimental methods, such as the primary, secondary, and tertiary structure of proteins. In order to utilize this wealth of protein data, numerous machine learning approaches have been designed to infer new PPIs. It is popular, among these approaches, to predict PPIs based on the structure of the protein information. For example, Agrawal et al. [11] proposed a computational tool—named a spatial interaction map (SIM)—that utilizes the structure of unbound proteins to detect the residues from PPIs. Qiu et al. [12] presented a novel residue characterization model, based on 3D structures, for the purpose of detecting PPIs. These computational methods—based on structural data—identify the interaction domain by analyzing the hydrophobicity, solvation, protrusion, and accessibility of residues. Since the volume of newly discovered protein sequence data is increasing exponentially, there is an increasingly larger gap between the volume of complex protein structure data, and that of protein sequence data [13,14]. Predicting PPIs based on structure data does not satisfy the requests of the many biochemists who have the sequences, but no structural data. Therefore, it is more important to develop effective computational models based on protein sequence data.

Currently, there are a number of different computational methods designed to implement this pattern in PPI prediction [15,16,17,18,19,20,21,22,23]. The common computational models for PPI prediction are composed of two key parts, namely, protein feature representation and sample classification. The purpose of the first step is to represent the proteins with useful attributes and transform the samples into feature vectors that are the same size as the sample classifier’s inputs. Effective feature descriptors can play an important role in improving the prediction performance of the system. 

Previous studies have shown that the evolutionary information on proteins may play a crucial role in predicting PPIs [24,25]. However, it is not easy to include evolutionary information in a protein sequence [26,27,28]. There is currently no single protein presentation method that takes full advantage of protein evolutionary information. Additionally, sequence evolution information is more difficult to use because of the differences in protein sequence length. In the face of such difficulties, how do we design a way to use the evolution information of proteins to implement the prediction of PPIs efficiently? In order to overcome this problem, we proposed a novel scheme that uses a position-specific scoring matrix (PSSM) to translate the protein sequence into a matrix, in which both the evolutionary information and the amino acid composition are included. Following this, we introduced a low-rank approximation (LRA) method to find the lowest level representation of all of the candidates and accurately recover the row space of the data to achieve high precision.

With regards to the second issue, some machine learning algorithms—such as random forests, neural networks, ensemble classifiers, random projections, and Naïve Bayes classifiers—are proposed for detecting PPIs to improve the accuracy of the prediction model [29,30,31]. The main trend in computational PPI detection is to achieve the highest precision, rather than speed, in the training of the classified model. Recently, relevance vector machines (RVMs) are a new statistical learning technique that provide the output of the probability classification, which uses Bayesian inferences to obtain a concise solution for the regression and classification [32]. Unlike support vector machines (SVM), RVM classifiers—with fewer input variables—provide better classification estimates for small, high dimensional datasets [33]. In this paper, the performances of RVMs and SVMs for classifying PPIs were compared. Using the PPI dataset, we show that the proposed method can quickly and effectively differentiate interactive protein pairs from large-scale data. The results of the experiment indicate that the proposed technique can complement experimental approaches for identifying PPI interactions.

In this paper, we proposed a novel protein representation method using protein evolutionary information. The main improvement was attributed to the use of LRA, a PSSM, and RVMs. In particular, we first used an LRA method on a PSSM that represented protein in a matrix form to obtain the feature vectors of the protein. Following this, the principal component analysis (PCA) method was employed to eliminate some of the noise and reduce the dimensions of the feature vectors. Finally, we used RVM classifiers to carry out the test. The proposed method was performed on the *Yeast* PPI dataset. The experimental results show that it is superior to SVM-based methods and other excellent technology that has been developed previously. Therefore, this approach is fit for predicting PPIs. Additionally, a user-friendly web server for predicting PPIs, PCLPred, was developed for academic users at http://219.219.62.123:8888/pclpred/.

The rest of this paper is organized as follows: Section 2 introduces the test results obtained from applying the proposed method, the SVM-based method, and several other existing methods. Section 3 describes the proposed approach. Section 4 summarizes the work presented in this paper. 

## 2. Results and Discussion

### 2.1. Five-Fold Cross-Validation

In this study, five-fold cross-validation methods were utilized to compare the performance of this model with other competing approaches. The whole PPI dataset is randomly divided into five roughly-equivalent subsets, each containing approximately equal amounts of interacting and non-interacting proteins. Four of the subsets are used for training and the remaining one is used for the test. This process is repeated five times, using a different subset of the test each time. The average of the five results is then calculated to ensure the highest level of fairness.

### 2.2. Comparison with the SVM-Based Approach Using the Same Feature Representation

In order to effectively assess the performance of the SVM classifier, we compared its performance with that of a state-of-the-art SVM classifier with the same feature extraction method on the *Yeast* dataset [34]. The LIBSVM (A Library for Support Vector Machines) tool provides an interface to facilitate the use of the SVM classifier. The cross-validation strategy is employed to optimize the related parameters of the SVM. Consequently, the parameters (c, g) are set to 0.8 and 0.4, respectively. Furthermore, the radial basis function is taken as the kernel function. 

**Figure 1:**
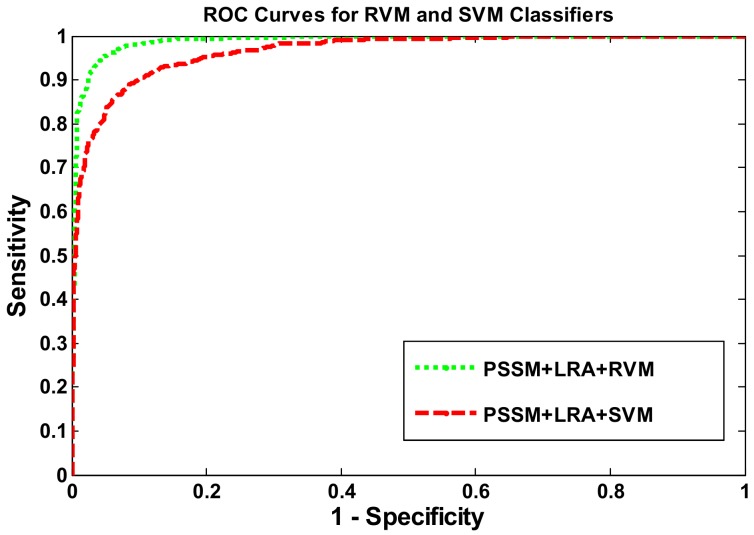
A comparison of the receiver operating characteristic (ROC) curves of the relevance vector machines (RVMs) classifier and the support vector machines (SVMs) classifier on the *Yeast* dataset.

The result of applying the two methods to the *Yeast* dataset are presented in Table 1, and the corresponding receiver operating characteristic (ROC) curves are shown in Figure 1. The prediction performance of the SVM classifier can be seen, from Table 1, to have achieved 89.4% accuracy, 88.5% sensitivity, 90.3% specificity, and 81.1% Matthews Correlation Coefficient (MCC). The average prediction results of applying the RVM classifier were 94.6% accuracy (which is 5.2% higher than the SVMs classifier) and 94.8% sensitivity (which is 6.3% higher than SVMs classifier). Several other indicators of the RVM classifier’s performance—shown in Table 1—are 4.0% above the performance of the SVM classifier. This comparison proves that the effect of using the RVM classifier to predict PPIs can be clearly distinguished from the effect of using the SVM classifier. Additionally, Figure 1 indicates that the ROC curves of the two classifiers also show that RVM classifier can be more powerful in detecting PPI performance than the SVM classifier. 

The reasons for this method producing better classification results come from the following points: (1) Based on a Bayesian framework to build a learning machine, the RVM classifier is conducive to making more scientific decisions based on the information; (2) in the choice of the kernel function, the RVM classifier is not limited by the Mercer theorem, and can construct any kernel function; (3) there is no need to set penalties. The penalty factor in the SVM classifier is a constant that balances the empirical risk and the confidence interval. The experimental results are very sensitive to the data. An improper setting may cause over-learning and other problems. The parameters in the RVM classifier, however, are automatically assigned; (4) compared to the SVM classifier, the RVM classifier is sparser, which means that the test time is shorter, making it more suitable for online testing. It is well known that the number of SVM support vectors grows linearly with the increase of the training samples, which is obviously not convenient when the training samples are very large. Although the RVM correlation vector also increases with the training samples, the growth rate is much slower than that of the SVM support vectors; and (5) previous research indicates that the RVM classifier has a better generalization performance than the SVM classifier. Additionally, when compared with the SVM classifier, the RVM classifier not only produces a binary output, but also gets the probability of the output.

### 2.3. A Comparison of the Proposed Method with Other Methods

Currently, many methods that are based on machine learning theory have been proposed for sequences-based PPIs. To assess the ability of the proposed approach, several existing techniques [35] are applied to the *Yeast* dataset and their results are compared to the results of our method. The comparison of the results of these methods is listed in Table 2. Table 2 clearly indicates that the proposed method achieved the highest average accuracy (94.6%) out of all of these methods. At the same time, the sensitivity and precision of the proposed technique are also superior to those of the other techniques. All of these results indicated that the RVM classifier, using the features vector that was extracted by the PSSM, LRA, and the PCA method, can substantially improve the quality of PPI prediction. This is mainly because of the efficient feature extraction strategy and the powerful classifier.

### 2.4. An Assessment of the Prediction Performance on the Helicobacter pylori PPI Dataset

In order to further investigate the prediction performance of our approach, we also compared the proposed approach with several other existing methods on the *Helicobacter pylori* PPI dataset. The prediction results for the abovementioned methods are reported in Table 3. In order to achieve a fair measure of randomness, we calculated the average of the measure values over five runs. We can observe from Table 3 that this method can achieve a good result, with 84.7% accuracy, 85.9% precision, and 84.4% sensitivity. It should be noticed that the precision and accuracy achieved by the proposed method are superior to those of the other methods. 

## 3. Materials and Methods

### 3.1. Dataset

In this paper, the proposed approach was verified on the high-confidence *Yeast* and *Helicobacter pylori* PPI datasets. We gathered the *Yeast* dataset from the publicly available Database of Interacting Proteins (DIP) [8]. For the purpose of ensuring the effectiveness of the experiment, we removed the protein pairs of less than fifty residues and greater than 40% sequence identity. By performing this screening work, the remaining 5594 protein pairs are reserved for building the positive dataset. The additional 5594 non-interacting protein pairs, with different subcellular localizations, were then used to build the negative dataset. As a result, the whole *Yeast* dataset finally consisted of 11,188 protein pairs. In order to further verify the general applicability of the proposed method, we also evaluated our method on the *Helicobacter pylori* PPI dataset. In total, we obtained 1458 positive samples and 1458 negative samples, as described by Martin et al. [39].

### 3.2. Position Specific Scoring Matrix (PSSM)

PSSM is a type of scoring matrix that was proposed by Gribskov et al. [24]. It is used to perform BLAST (Basic Local Alignment Search Tool) searches, where amino acid substitution scores are assigned to a specific location in the proteins’ multiple sequence alignments. It has been successfully applied in various fields of biological information because it contains the evolutionary information of proteins. PSSM is represented as a *T ×* 20 matrix that can be interpreted as $M=\left\{c_{i,j}:i=1\cdots T\;\mathrm{and}\;j=1\cdots 20\right\}$. The representation of PSSM is as follows:

(1) $$M=\left[\begin{array}{c} \begin{array}{cc} c_{1,1} & c_{1,2} \end{array}\;\begin{array}{cc} \cdots & c_{1,20} \end{array}\\ \begin{array}{cc} c_{2,1} & c_{2,2} \end{array}\;\begin{array}{cc} \cdots & c_{2,20} \end{array}\\ \begin{array}{cc} \vdots \; & \vdots \; \end{array}\;\begin{array}{cc} \vdots \; & \vdots \end{array}\\ \begin{array}{cc} c_{L,1} & c_{L,2} \end{array}\;\begin{array}{cc} \cdots & c_{L,20} \end{array} \end{array}\right]$$

The elements in this matrix are generally expressed as integers (negative or positive). A higher score indicates that a given amino acid substitution occurs frequently in the alignment, while a lower score indicates a lower frequency of the substitution.

We created the PSSM using a Position-Specific Iterated BLAST (PSI-BLAST, Bethesda, MD, USA), which found a protein sequence that was similar to the query sequence, and then constructed the PSSM from the obtained alignment. In this work, we set the number of iterations to three and the *e*-value to 0.001 and *t*, respectively, in order to obtain a highly broad homologous sequence.

### 3.3. Low-Rank Approximation (LRA)

LRA is a widely used method for matrix analysis, where the cost function measures the fit between an approximation matrix (optimization variable) and a given sparse matrix, constrained by the reduced rank of the approximation matrix [40,41]. In this case, using LRA on the PSSM of the obtained protein sequences results in a descriptor containing evolutionary information that is used for representing a protein. For a 20 × *L* feature matrix *N*, the LRA would be written as follows:

(2) $$\underset{\hat{N}}{\min}{\left\|N-\hat{N}\right\|}_{F}$$

(3) $$\mathrm{Subject}\;\mathrm{to}:\;rank(\hat{N})\leq r$$

where ${\left\|•\right\|}_{F}$ represents the Frobenius norm. Formula (2) is solved using the singular value decomposition (SVD) method.

Let $N=U\sum V^{T}\in R^{m\times n}$ be the SVD of *N* and partition *U*, $\sum =:diag(\sigma_{1},\sigma_{2},\sigma_{3},\ldots ,\sigma_{20})$, and *V* as follows:

(4) $$U=:\left[\begin{array}{cc} U_{1} & U_{2} \end{array}\right],\;\sum =:[\begin{array}{cc} \sum_{1} & 0\\ 0 & \sum_{2} \end{array}],\;\mathrm{and}\;V=:[\begin{array}{cc} V_{1} & V_{2} \end{array}]$$

where $\sum_{1}$ is a square array of *r*. *U*_1_ and *V*_1_ represent different matrices, and their sizes are *m* × *r* and *n* × *r*. The rank-*r* matrix can then be gained as follows:

(5) $${\hat{N}}^{*}=U_{1}\sum_{1}V_{1}{}^{T}$$

where ${\left\|N-{\hat{N}}^{*}\right\|}_{F}=\underset{rank(\hat{N})\leq r}{\min}{\left\|N-\hat{N}\right\|}_{F}=\sqrt{\sigma_{r+1}^{2}+\sigma_{r+2}^{2}+\ldots +\sigma_{m}^{2}}$.

The Σ112, with dimensions *r*-by-*r,* can be obtained by computing the square root of the reduced matrix $\Sigma_{1}$, in which the sequence order information of the protein is contained. It is noteworthy that the feature matrix *N* of the protein may have a different number of columns, which is caused by the unequal lengths of protein sequences. However, the U1Σ112 is a fixed length (a 20 × *r* matrix).

We form a vector from the gained matrix U1Σ112 by concatenating all of the rows, from row 1 to 20, of matrix U1Σ112. Therefore, the feature descriptor consists of a total of 20 × *r* descriptor values. Considering the trade-off between the cost of computing for extracting the protein feature and the overall prediction accuracy, the optimal rank is 5. We connect the descriptors of the two protein sequences to represent an interaction pair. 

### 3.4. Properties of the Proposed Algorithm

Based on orthogonal triangular decomposition theory and LRA theory, the properties of the PSSM feature extraction algorithm are deduced.

**Lemma** **1.** Suppose that matrix ${\hat{N}}^{*}$ in (5) satisfies (3). For the Frobenius norm, if r≤rank(N), then ${\hat{N}}^{*}$ is unique if and only if N’s rth and (r+1)th largest singular values differ.

**Proof.**  ${\hat{N}}^{*}$ is a solution to

(6) $${\hat{N}}^{*}:=\arg\underset{\hat{M}}{\min}{\left\|N-\hat{N}\right\|}_{F}$$

$$s.t.\;\;\;\;\;\;\;\;rank(\hat{N})\leq r.$$

and ${\hat{N}}^{*}:=U^{*}\sum^{*}{\left(V^{*}\right)}^{T}$ is an SVD of ${\hat{N}}^{*}$. Based on the single invariance of the Frobenius norm, we have

(7) $${\left\|N-{\hat{N}}^{*}\right\|}_{F}={\left\|{\left(U^{*}\right)}^{T}(N-{\hat{N}}^{*})V^{*}\right\|}_{F}={\left\|{\left(U^{*}\right)}^{T}NV^{*}-\sum^{*}\right\|}_{F}$$

where ${\left(U^{*}\right)}^{T}NV^{*}=\hat{N}$. Partition

(8) $$\hat{N}=\left[\begin{array}{cc} {\hat{N}}_{11} & {\hat{N}}_{12}\\ {\hat{N}}_{21} & {\hat{N}}_{22} \end{array}\right]$$

conformably with $\sum^{*}=\left[\begin{array}{cc} \sum^{*}{}_{1} & 0\\ 0 & 0 \end{array}\right]$ and observe that

(9) $$rank\left(\left[\begin{array}{cc} \sum^{*}{}_{1} & {\hat{N}}_{12}\\ 0 & 0 \end{array}\right]\right)\leq m\;\;\;\;\;\mathrm{and}\;\;\;\;\;{\hat{N}}_{12}\neq 0\Rightarrow {\left\|\hat{N}-\left[\begin{array}{cc} \sum^{*}{}_{1} & {\hat{N}}_{12}\\ 0 & 0 \end{array}\right]\right\|}_{F}<{\left\|\hat{N}-\left[\begin{array}{cc} \sum^{*}{}_{1} & 0\\ 0 & 0 \end{array}\right]\right\|}_{F}$$

Thus, ${\hat{N}}_{12}=0$. Similarly, ${\hat{N}}_{21}=0$. Observe also that

(10) $$rank\left(\left[\begin{array}{cc} {\hat{N}}_{11} & 0\\ 0 & 0 \end{array}\right]\right)\leq m\;\;\;\;\;\;\mathrm{and}\;{\hat{N}}_{11}\neq \sum_{1}{}^{*}\Rightarrow {\left\|\hat{N}-\left[\begin{array}{cc} {\hat{N}}_{11} & 0\\ 0 & 0 \end{array}\right]\right\|}_{F}<{\left\|\hat{N}-\left[\begin{array}{cc} \sum^{*}{}_{1} & 0\\ 0 & 0 \end{array}\right]\right\|}_{F}$$

Thus, ${\hat{N}}_{11}=\sum_{1}^{*}$. Therefore,

(11) $$\hat{N}=\left[\begin{array}{cc} \sum^{*}{}_{1} & 0\\ 0 & {\hat{N}}_{22} \end{array}\right]$$

Let ${\hat{N}}_{22}=U_{22}\sum_{22}V_{22}{}^{T}$ be the SVD of ${\hat{N}}_{22}$. Then the matrix

(12) $$\left[\begin{array}{cc} I & 0\\ 0 & U_{22}{}^{T} \end{array}\right]\hat{N}\left[\begin{array}{cc} I & 0\\ 0 & V_{22} \end{array}\right]=\left[\begin{array}{cc} \sum_{1}^{*} & 0\\ 0 & \sum_{22} \end{array}\right]$$

has the optimal rank-*m* approximation $\sum^{*}=\left[\begin{array}{cc} \sum_{1}^{*} & 0\\ 0 & 0 \end{array}\right]$, such that

(13) $$\min(diag(\sum_{1}^{*}))>\max(diag(\sum_{22}))$$

Therefore,

$N=U^{*}\left[\begin{array}{cc} I & 0\\ 0 & U_{22} \end{array}\right]\left[\begin{array}{cc} \sum_{1}^{*} & 0\\ 0 & \sum_{22} \end{array}\right]\left[\begin{array}{cc} I & 0\\ 0 & V_{22}{}^{T} \end{array}\right]{\left(V^{*}\right)}^{T}$ is an SVD of *N*.

Thus, if $\sigma_{m}>\sigma_{m+1}$, the rank-*m* truncated SVD

(14) $${\hat{N}}^{*}=U^{*}\left[\begin{array}{cc} \sum_{1}^{*} & 0\\ 0 & \sum_{22} \end{array}\right]{\left(V^{*}\right)}^{T}=U^{*}\left[\begin{array}{cc} I & 0\\ 0 & U_{22} \end{array}\right]\left[\begin{array}{cc} \sum_{1}^{*} & 0\\ 0 & 0 \end{array}\right]\left[\begin{array}{cc} I & 0\\ 0 & V_{22}{}^{T} \end{array}\right]{\left(V^{*}\right)}^{T}$$

is unique and ${\hat{N}}^{*}$ is the unique solution of LRA. □

The salient feature of Lemma 1 is that, although the rank constraint is highly non-convex and non-linear, one is still able to efficiently solve (2) using the SVD method. Additionally, under all of the consistent rules, there is an optimal solution under the *Frobenius* norm.

### 3.5. Relevance Vector Machine (RVM) Model

The RVM model is a probabilistic model under a Bayesian framework, developed by Tipping et al. [32,33,42]. It has been widely applied for solving classification and regression problems. Assuming that the training datasets are (*x_n_*,*y_n_*)*_n_*_=1_*^N^* for binary classification problems, $x_{n}\in R^{d}$ is the training sample; $t_{n}\in \left(0,1\right)$ denotes the label of the training dataset; $t_{i}$ is the label of the testing dataset; $t_{i}=b_{i}+\varepsilon_{i}$, where $b_{i}=w^{T}\varphi \left(x_{i}\right)=\sum_{j=1}^{N}w_{j}K\left(x_{i},x_{j}\right)+w_{0}$, is the classification model; and $\varepsilon_{i}$ is the additional noise, with a variance of $\sigma^{2}$ and a mean value of zero, where $\varepsilon_{i}\sim N\left(0,\sigma^{2}\right),y_{i}\sim N\left(b_{i},\sigma^{2}\right)$. The training datasets are assumed to be independent and distributed identically. The observation of vector t follows the distribution as follows:

(15) $$m\left(y|x,c,\sigma^{2}\right)={\left(2\pi \sigma^{2}\right)}^{-N/2}\exp[-\frac{1}{2\sigma^{2}}{\left|\left|y-\partial c\right|\right|}^{2}]$$

where ∂ meets the following definition:

(16) $$\partial =\left(\begin{array}{ccc} 1 & k\left(x_{1},x_{1}\right)\cdots & k\left(x_{1},x_{N}\right))\\ \ldots & \ldots & \ldots \\ 1 & k\left(x_{N},x_{1}\right)\ldots & k\left(x,x_{N}\right) \end{array}\right)$$

The method used by the RVMs to predict the label *t* * of a test sample is given by:

(17) $$m\left(y_{*}|y\right)=\int^{\text{​}}p\left(y_{*}|c,\sigma^{2}\right)p(c,\sigma^{2}|y)dwd\sigma^{2}$$

In order to reduce the computational complexity of the kernel function and ensure that the majority of the weight vector has a value of zero, the weight vector *w* is limited by extra conditions. Assuming that $c_{i}\sim N\left(0,\;x_{i}^{-1}\right)$, $p\left(w|x\right)=\prod_{i=0}^{N}p(c_{i}|x_{i})$, where x is a hyper-parameters vector.

(18) $$m\left(t_{*}|t\right)=\int^{\text{​}}p\left(t_{*}|w,x,\sigma^{2}\right)p(c,x,\sigma^{2}|t)dwdad\sigma^{2}$$

(19) $$m\left(t_{*}|c,x,\sigma^{2}\right)=N(t_{*}\;|b\left(a_{*};c\right),\sigma^{2})$$

We get $p\left(c,x,\sigma^{2}|t\right)$ using the Bayesian formula

(20) $$m\left(c,x,\sigma^{2}|t\right)=p(x,\sigma^{2}|t)p\left(x|x,\sigma^{2},t\right)$$

(21) $$m\left(c|x,\sigma^{2},t\right)=p\left(t|c,\sigma^{2}\right)p\left(c|x\right)/p\left(t|x,\sigma^{2}\right)$$

The integral of the product of $p(t|x,\sigma^{2})$ and p(c|x) is given by

(22) $$m\left(t|x,\sigma^{2}\right)={\left(2\pi \right)}^{-N/2}{\left|\Omega \right|}^{-1/2}\exp\left(-\frac{t^{T}\Omega^{-1}t}{2}\right)$$

(23) $$\Omega =\sigma^{2}I+\partial A^{-1}\partial^{T},\;A=diag\left(x_{0,}x_{1,}\ldots ,x_{N}\right)$$

(24) $$m\left(c|x,\sigma^{2},t\right)={\left(2\pi \right)}^{-\left(N+1\right)/2}{\left|\Sigma \right|}^{-1/2}\exp\left(-\frac{{\left(c-u\right)}^{T}\left(c-u\right)}{2}\right)$$

(25) $$\Sigma ={\left(\sigma^{-2}\partial^{T}\partial +A\right)}^{-1}$$

(26) $$u=\sigma^{-2}\Sigma \partial^{T}t$$

The maximum likelihood method was used to solve $m(x,\sigma^{2}|t$)$\propto m\left(t|x,\sigma^{2}\right)m\left(x\right)m\left(\sigma^{2}\right)$ and $\;m(x,\sigma^{2}|t$), and is represented by

(27) $$\left(x_{MP},\sigma_{MP}^{2}\right)=\underset{\;x,\sigma^{2}}{\arg}maxp(t|x,\sigma^{2})$$

The iterative process of $x_{MP}$ and $\sigma_{MP}^{2}$ is as follows:

(28) $$\{\begin{array}{c} x_{i}^{new}=\frac{\gamma_{i}}{\mu_{i}^{2}}\\ {\left(\sigma^{2}\right)}^{new}=\frac{{\left|\left|t-\partial \mu \right|\right|}^{2}}{N-\sum_{i=0}^{N}\mu_{i}}\\ \gamma_{i}=1-x_{i}\sum^{\text{​}}i,i \end{array}$$

where $\sum^{\text{​}}i,i$ represents the ith element on the diagonal of Σ, and the initial value of a and $\sigma^{2}$ are determined via the approximation of $a_{MP}$ and $\sigma_{MP}^{2}$, by continuously using Formula (19). 

### 3.6. Procedure of the Proposed Method

**Figure 2:**
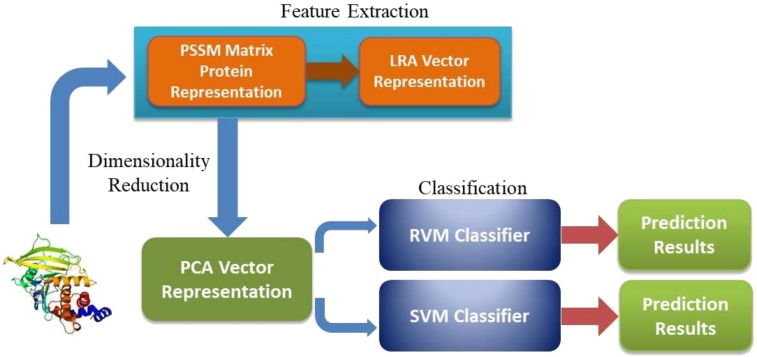
The flow chart of the proposed method.

In the study, the workflow of the PCLPred method is presented in Figure 2. More specifically, the protein amino acids sequence datasets are downloaded from DIP. The CD-HIT (Cluster Database at High Identity with Tolerance) and PSI-BLAST programs are then used to remove sequence redundancy and generate PSSM, respectively [43]. Following this, LRA is employed to obtain the feature representation from PSSM, which contains a large volume of valuable evolutionary knowledge for PPI prediction. After the dimensionality reduction—using the PCA technique—the significant features are extracted and used as input features to train the RVM classifier. Finally, the prediction performance is evaluated using five-fold cross-validations [44,45,46,47,48].

### 3.7. Performance Evaluation

In order to evaluate the performance of the designed model, a number of validation measures are employed.

(1) Overall prediction accuracy:

(29) $$Accuracy=\frac{T_{P}^{}+T_{N}^{}}{T_{P}+F_{P}+T_{N}+F_{N}}$$

(2) Sensitivity:

(30) $$Sensitivity=\frac{T_{P}}{T_{P}+F_{N}}$$

(3) Specificity:

(31) $$Specificity=\frac{T_{N}}{T_{N}+F_{P}}$$

(4) Positive predictive value:

(32) $$PPV=\frac{T_{P}}{T_{P}+F_{P}}$$

(5) Negative predictive value:

(33) $$NPV=\frac{T_{N}}{T_{N}+F_{N}}$$

(6) *F*-score:

(34) $$F_{s}=2\times \frac{Sen\times PPV}{Sen+PPV}$$

(7) Matthews correlation coefficient:

(35) $$MCC=\frac{T_{P}\times T_{N}-F_{P}\times F_{N}}{\sqrt{\left(T_{P}+F_{N})\times (T_{N}+F_{P})\times (T_{P}+F_{P})\times (T_{N}+F_{N}\right)}}$$

where *T_P_* is true positive, indicating that the total number of interactive proteins will be predicted correctly; *F_P_* is false positive, indicating the total number of these proteins pairs that have no interaction, but are determined as interacting; *F_N_* is false negative, indicating the total number of interactive proteins that are determined as non-interacting; and *T_N_* is true negative, indicating the total number of these proteins pairs that have no interaction that are determined correctly. Additionally, the ROC curve is adopted as a measure that is used to evaluate the prediction performance of the different methods [49,50].

## 4. Conclusions

In this study, we proposed a novel computation-based automated decision-making method by employing the RVM model combined with the LRA method and PSSM. More specifically, LRA is employed to obtain the feature representation from PSSM, which contains a large volume of valuable evolutionary knowledge for PPI prediction. The RVM classifier is then applied to predict novel PPIs. Extensive computational experiments are performed on several PPI datasets in order to evaluate the PPI identification ability of the developed approach. These experimental results have proven that the PPI identification ability of this approach is clearly stronger than that of the SVM-based method and several other existing approaches. The promising results demonstrate that the proposed method is an efficient and reliable approach to detecting PPIs. It is also a practical tool that will help to advance research in the field of bioinformatics.

## Figures and Tables

**Table 1:** Five-fold cross-validation results shown using our proposed method on the *Yeast* dataset.

Model	Testing Set	Accuracy	Sensitivity	Specificity	PPV	NPV	MCC
PSSM+ LR+RVM	1	94.7%	95.4%	94.0%	93.9%	95.46%	89.3%
	2	95.3%	96.1%	94.5%	94.7%	95.96%	91.1%
	3	93.9%	93.9%	93.8%	93.8%	93.91%	88.5%
	4	93.8%	93.6%	94.1%	94.4%	93.22%	88.4%
	5	95.1%	94.9%	95.2%	94.9%	95.2%	90.6%
	Average	**94.6 ± 0.6%**	**94.8 ± 1.0%**	**94.3 ± 0.5%**	**94.3 ± 0.4%**	**94.75 ± 1.1%**	**89.6 ± 1.2%**
PSSM+ LR+SVM	1	88.3%	87.3%	89.3%	88.8%	87.8%	79.4%
	2	89.3%	89.4%	89.1%	89.2%	89.3%	80.8%
	3	89.8%	89.2%	90.3%	90.7%	88.8%	81.6%
	4	89.7%	88.3%	91.2%	90.9%	88.6%	81.6%
	5	90.0%	88.4%	91.5%	90.8%	89.2%	81.9%
	Average	**89.4 ± 0.6%**	**88.5 ± 0.8%**	**90.3 ± 1.0%**	**90.1 ± 1.0%**	**88.7 ± 0.5%**	**81.1 ± 1.0%**

SVM: support vector machine; PSSM: position specific scoring matrix; AB: average blocks; RVM: relevance vector machine; PPV: Positive Predictive Value; NPV: Negative Predictive Value; MCC: Matthews Correlation Coefficient.

**Table 2:** The prediction ability of the different methods on the *Yeast* dataset.

Model	Testing Set	Acc (%)	Sen (%)	Pre (%)	Mcc (%)
Guos’ work [35]	ACC	89.3 ± 2.6	89.9 ± 3.6	88.8 ± 6.1	N/A
	AC	87.4 ± 1.3	87.3 ± 4.6	87.8 ± 4.3	N/A
Zhous’ work [36]	SVM+LD	88.6 ± 0.3	87.4 ± 0.2	89.5 ± 0.6	77.2 ± 0.7
Yangs’ work [37]	Cod1	75.1 ± 1.1	75.8 ± 1.2	74.8 ± 1.2	N/A
	Cod2	80.0 ± 1.0	76.8 ± 0.6	82.2 ± 1.3	N/A
	Cod3	80.4 ± 0.4	78.1 ± 0.9	81.7 ± 0.9	N/A
	Cod4	86.2 ± 1.1	81.0 ± 1.7	90.2 ± 1.3	N/A
Yous’ work [38]	PCA-EELM	87.0 ± 0.2	86.2 ± 0.4	87.6 ± 0.3	77.4 ± 0.4
Proposed method	LRA+RVM	94.6 ± 0.6	94.8 ± 1.0	94.4 ± 0.4	89.6 ± 1.2

ACC: Auto Covariance; LD: Local Description; PCA: Principal Component Analysis; EELM: Ensemble Extreme Learning Machines; N/A: Not Available; Acc: Accuracy; Sen: sensitivity; Pre: precision; Mcc: Matthew’s Correlation Coefficient.

**Table 3:** The prediction ability of the different methods on the *Helicobacter pylori* protein–protein interactions (PPIs) dataset.

Methods	Acc(%)	Sen (%)	Pre (%)	Mcc (%)
HKNN	84.0	86.0	84.0	N/A
Phylogenetic bootstrap	75.8	69.8	80.2	N/A
Signature Products	83.4	79.9	85.7	N/A
Boosting	79.5	80.4	81.7	N/A
Proposed method	**84.7 ± 1.0**	**84.4 ± 1.2**	**85.9 ± 0.8**	**76.7 ± 1.0**

HKNN: Hyperplane Distance Nearest Neighbor.
